# Supplementary material for: Regional clozapine, ECT and lithium usage inversely associated with excess suicide rates in male adolescents
Source: Nat Commun. 2023 Mar 14;14:1281. doi: 10.1038/s41467-023-36973-4 (PMC10015020; doi:10.1038/s41467-023-36973-4)
Supplement: Supplementary file 1 — Supplementary Information [file 41467_2023_36973_MOESM1_ESM.pdf]

Supplementary Material – Supplementary Methods, Figures, Supplementary Tables (R output)

Supplementary Methods

2.1 Data sources and initial processing

Openly available data from the Swedish National Board of Health and Welfare (available in Swedish: https://sdb.socialstyrelsen.se/if_dor/val.aspx; https://sdb.socialstyrelsen.se/if_lak/val.aspx) was extracted for 21 Swedish counties across 2016-2020 in the age-ranges 15-19 and 20-24, respectively, and for the following variables: The number of dispensations to adolescents recorded for lithium (ATC-code N05AN01) and clozapine (ATC-code N05AH02) per 1,000 inhabitants (based on population estimates from January 1st of the recorded year), and confirmed suicide death rates per 100,000 inhabitants. All data was extracted for both sexes, males, and females, respectively - across all Swedish counties for the years 2016-2020. Values for lithium and clozapine were divided by 100 to arrive at a representative estimate per 100,000 inhabitants. For transparency, data on the number of dispensations for lithium and clozapine are available from 2006-2021 and rates of suicide deaths from 1997-2020. Moreover, data in the age-ranges 0-17 was only available at an aggregate level, and, hence, not allowing for the distinction of clozapine from other neuroleptics or detailing lithium dispensations. Data for the years 1997-2015 for the age-ranges 0-17 was not analyzed (rationale presented in part above and below). Suicide death rates was not available for the year 2021. Thus, this year was not extracted. Similarly, openly available data from the Swedish ECT registry (available in Swedish: https://ect.registercentrum.se/statistik/utdata-ect/p/rJ3vhF3Lw) detailing the number of patients receiving ECT treatment (age-group and sex) was extracted for the years 2016-2020 and each region in the age-ranges 0-17 (<18). For transparency, ECT treatment frequencies were available in the age-ranges 0-17 (<18) and 18-39 (and in higher ages). The number of ECT treatments in the 0-17 age-range was manually extracted for each year and region, separately, and adjusted to population estimates from January 1st of the recorded year – to arrive at a representative estimate of ECT treatment usage frequencies per 100,000 inhabitants. Input data alongside detailed calculations are available as Supplementary Material.

2.2 Statistical considerations

Prior to statistical association analyses, several measures were taken with the aim of strengthening robustness of included variables. These initial steps were performed in using Microsoft Excel for Microsoft 365 MSO (Version 2204 Build 16.0.15128.20278). (**1**) Initially, the mean and standard deviation was compared for each key variable across 2016 to 2020 in both sexes, males, and females – measured per 100,000 inhabitants. Several counties exhibited greater standard deviation than mean value for key vari ables, i.e., for clozapine (both sexes, n=9; females, n=9; males, n=9), lithium (both sexes, n=2; females, n=4; males, n=5), ECT (both sexes, n=15; females, n=9; males, n=8), baseline adolescent suicide rates (both sexes, n=7; females, n=11; males, n=10) and baseline suicide rates in young adulthood (both sexes, n=2; females, n=13; males, n=5) – overall implicating that the median value across 2016 to 2020 would be more statistically appropriate as compared to the mean. However, it was noted that several counties provided certain treatments or exhibited adolescent suicide deaths only for one to two of the five years i.e., for clozapine (both sexes, n=4; females, n=4; males, n=6), lithium (both sexes, n=0; females, n=1; males, n=3), ECT (both sexes, n=9; females, n=6; males, n=5), baseline adolescent suicide rates (both sexes, n=5 females, n=10; males, n=8) and baseline adult suicide rates (both sexes, n=1; females, n=11; males, n=2). Hence, both mean and median values were considered inappropriate to adequately represent treatment usage frequencies and suicide death rates across all counties in the years 2016-2020. Therefore, min-max normalization was implemented across each year, region, and treatment type (i.e., for example, $DerivedValue_{2016}$ = $Value_{2016}$-min($Value_{2016-2020}$)/(max($Value_{2016-2020}$)-min($Value_{2016-2020}$))). In the case of suicide death rates, the difference in suicide rates in adolescence and young adulthood per 100,000 inhabitants was calculated for each region, year and sex group (i.e., $DerivedExcessSuicidRateAdolescence_{2016}$ = $SuicideRateAdolescence_{2016}$ - $SuicideRateYoungAdulthood_{2016}$)). Thus, one excess-suicide-rate value was derived for each region, year, and sex group (both sexes, females, and males, respectively). Subsequently, this value was subjected to the same min-max normalization procedure as described above. In cases of zero-value denominators, derived values were manually inserted as zero. The average min-max normalized values across 2016-2020 was implemented in the subsequent analysis, whereby each region and sex-group was represented by a mean treatment value measured across

2016-2020 and one value for excess adolescent suicide deaths. When compared to the median derived values across 2016-2020, the mean value was considered more representative of the underlying data distribution by, for example, also recognizing counties providing treatment in only one or two years in the studied five-year-period. Derived values were visually inspected across all key variables and sex-groups and deemed overall representative of the underlying data (**Supplementary Table 1.**). (**2**) To reduce effects of potential unmeasured sources of confound on suicide rates across counties, the primary outcome variables were based on subtraction between suicide death rated in adolescence and young adulthood. Thus, the influence of regional differences on suicide death rates in, for example, population size, socioeconomic status, substance abuse, or availability and quality of psychiatric care, could thus largely be reduced. Similarly, subsequent min-max normalization contributed to normalizing potential confounding effects of substantial outliers regarding baseline absolute values. (**3**) To ensure measurements for lithium and clozapine correspond to long-term usage, extracted data consisted of the number of dispensations. Thus, any patients receiving treatment for a short-time period (for example during inpatient treatment) – arguably not reflective of adequate treatment - would exert less influence compared to patients consistently provided with such prescriptions. This was not possible in the case of ECT, as the number of ECT treatments received was not openly available. Thus, instead the total number of patients receiving ECT treatment was implemented. Moreover, large fluctuations in advanced treatment usage were not considered conciliable with best practice care. Especially so in the case of lithium and clozapine where, for example, dramatic reductions in the number of dispensations (sometimes to zero) could indicate that all treated patients either transitioned into a different age-group or, more probably, conferred by a substantial portion of patients being discontinued from such advanced treatments. Thus, min-max normalization contributed to downregulating potentially confounding treatment values from regions exhibiting one or two years with extremely high values and zero (no treatment) for the other years. (**4**). The time-period was chosen to match the major course direction in national treatment guidelines regarding ECT – since 2016 and with highest priority recommending its use in the care of post-pubertal adolescents with severe MDD with mood-congruent psychotic symptoms, catatonia, or treatment resistance. Similarly, information on regional suicide death rates was available up to 2020, hence, 2021–2022 treatment frequencies were not included in the analysis (**5**). Moreover, confounding could arise from the putative general underutilization of ECT for minors – i.e., according to the Swedish National Quality Registry for ECT

(coverage rate ~90%) fewer than 20 adolescents in Sweden received ECT treatment in 2020[1], encompassing a population exceeding 10 million. Aversiveness to brain stimulation techniques could thus mask the adequate recognition of severe mental illness, in the case of clinicians adequately implementing lithium and clozapine on correct indication but withholding the option of ECT-treatment from adolescent populations. Thus, mean usage frequencies across the min-max normalized 2016-2020 values (derived in Section 2.1 (1) was implemented for the subsequent analysis in the case of ECT (ages 13-17), lithium and clozapine (both ages 15-19). These measures should further increase robustness, while also allowing for the recognition of ECT-aversive (or Clozapine or Lithium-aversive) counties that may provide adolescents with adequate recognition of severe mental illness (and treatment with other studied modalities when indicated). (**7**) Adolescent treatment usage frequencies were generally of small magnitude across counties. Hence, it cannot be completely excluded that the influence of a single clinician in a medium-sized region would significantly alter measured frequencies – while possibly unrepresentative of the care provided in the full region. To reduce the influence of any outlier counties regarding frequency of treatment usage, we implemented robust linear regression models. Lastly, (**8**) we recognized the large impact that a single suicide attempt or ECT-treatment could yield in a small to medium-sized region, potentially exerting disproportionate confounding effects on downstream analyses. Therefore, additional weights were added to the robust models based on regional population in relation to total population (2020 estimates) – reducing potential unbalanced effects from outlier counties with small populations.

2.2 Statistical analysis

Initial data processing (i.e., calculation of min-max normalized values) was performed using Microsoft Excel365 MSO [Version 2210 Build 16.0.15726.20188] 64-bit). All downstream statistical analyzes were performed using R version 4.0.3. The variables included in the analysis pertained to mean usage frequencies across the min-max normalized 2016-2020 values for ECT (ages 13-17), lithium and clozapine (both ages 15-19); and the min-max normalized differential suicide death rate between adolescence and young adulthood. A total of 21 Swedish counties were included – each with three proxy values for clozapine-ECT-lithium usage frequencies (both sexes, females, and males, respectively) and three proxy values for excess adolescent suicide rates (both sexes, females, and

males, respectively). The Shapiro–Wilk normality test indicated that all included (six) variables satisfied requirements for normal distribution ($p>0.1$)(**Supplementary Figure 1.**). The relative contribution of each treatment modality to the averaged treatment proxy variable were assessed by comparing coefficients by multiple ordinary least squares regression ($Proxy_{2016\text{-}2020} \sim Clozapine_{2016\text{-}2020} + ECT_{2016\text{-}2020} + Lithium_{2016\text{-}2020}$) – performed separately for each sex category (both sexes, males, and females, respectively). Associations across counties between excess suicide death rates in adolescence and mean treatment usage frequencies by robust linear regression models using the R–packages 'robustbase'[2] and 'rcompanion'[3], specifying recommended setting (KS2014) – performed separately for both sexes, males and females, respectively[4]. Chain of regression estimates included the standard MM-regression estimator (guaranteeing an acceptable compromise between high breakdown (i.e., 50%) and very high efficiency (i.e., 95%)[5]) – while adjusting for weights (2020 regional population in proportion to national estimates the same year) and using the recommended setting (KS2014). Models exhibiting p-values for the primary explanatory variable < 0.05 were considered significant. Main models were illustrated by x–y scatterplots using the R-package 'ggplot2'[6], with the estimated slope coefficient from the regression model (**Figure 1.**). As a final validation step of significant models, we tested the hypothesis whether counties in the lower quartile (Q1) regarding excess adolescent suicide deaths exhibited greater advanced treatment usage frequencies in adolescents compared to Q2-Q4 counties. The excess adolescent suicide death rate variable was thus dichotomized by the 25th quartile and contrasted to the estimate of advanced treatment usage frequencies by the one-sided Wilcoxon rank sum exact test. P–values < 0.05 were considered significant and these models were illustrated by dotplot boxplots using the R-package 'ggplot2'[6]. An openly available code originally posted by Laura DeCicco was further implemented to provide a more detailed complementary legend plot[7]. Post-hoc analyses were subsequently performed to determine the effects of any single treatment modality. First, the min-max normalized values across 2016-2020 for clozapine, ECT and lithium, respectively, were assessed by the Shapiro Wilk's test – indicating non-normal distribution of values for clozapine and ECT across all investigated sex groups ($p<0.05$), thus subjected to transformation by Blom's method[8] for subsequent robust linear regression analyses (but not for nonparametric tests, i.e., the Wilcoxon rank sum exact test). To minimize putative bias from collinearity on downstream analyzes, the mean of treatment variables that were strongly correlated according to the Pearson's Correlation Coefficient (i.e., $r>0.5$) was

calculated, tested for normal distribution by Shapiro Wilk's tests and implemented in subsequent analyses (i.e., ECT and lithium for the combined sex group, and clozapine and lithium for the male subgroup). Thereafter, robust linear regression models (same specifications as in the main model) were implemented, contrasting excess adolescent suicide death rates to these variables, separately for each sex group (i.e., for example - $SuicideRates_{2016\text{-}2020}$~$Clozapine_{2016\text{-}2020}$+Mean($ECT_{2016\text{-}2020}$ + $Lithium_{2016\text{-}2020}$) - for the combined sexes group). To reduce potential bias from overfitting of the model (given the small sample size, n=21 regions), treatment variables were tested separately for the female subgroup – and resulting significance values were subjected to stringent Bonferroni-correction[9]. As in the main analysis, significant associations were validated by contrasting the dichotomized 25th quartile based on excess suicide death rates to the candidate treatment variable. In these post-hoc analyses of non-Blom-transformed values, p–values < 0.05 (Bonferroni-adjusted in the case of females) were considered significant.

Supplementary Figure 1. Histograms of Key Variables (Excess Suicide Death Rates and Mean Advanced Treatment Usage Frequencies)

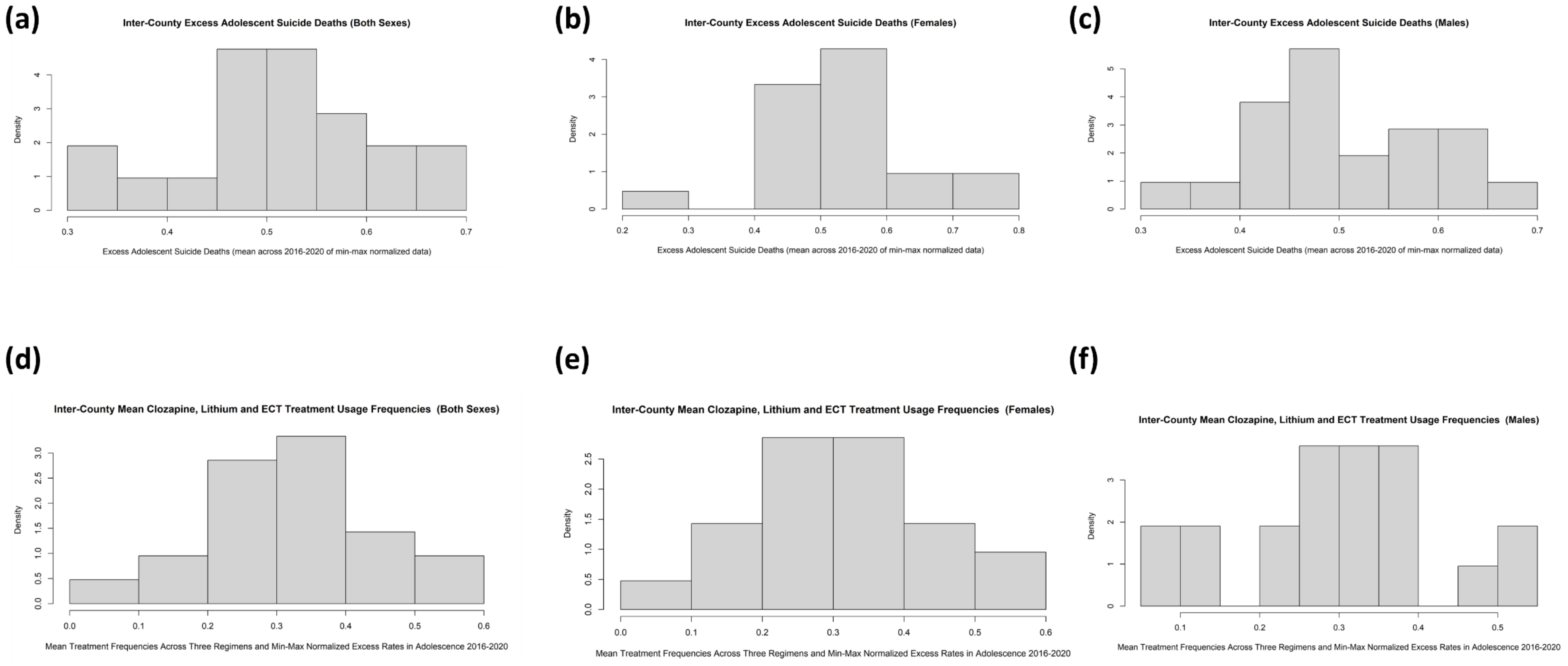

Legend (Supplementary Figure 1): Histograms of mean min-max normalized values across 2016-2020 (for (e-f) also mean across clozapine, ECT and lithium treatments). a-c corresponds to values used in the analysis for regional excess adolescent suicide deaths in both sexes (a), females (b) and males (c). Similarly, d-f represents values for

regional mean clozapine, lithium and ECT treatment usage frequencies in both sexes (d), females (e) and males (f). All variables were normally distributed according to the Shapiro Wilk's Normality Test ($p>0.1$).

Supplementary Figure 2. Dotplot Boxplots Depicting ECT and Lithium Usage Frequencies and Regional Excess Suicide Deaths across Both Genders (2016-2020)

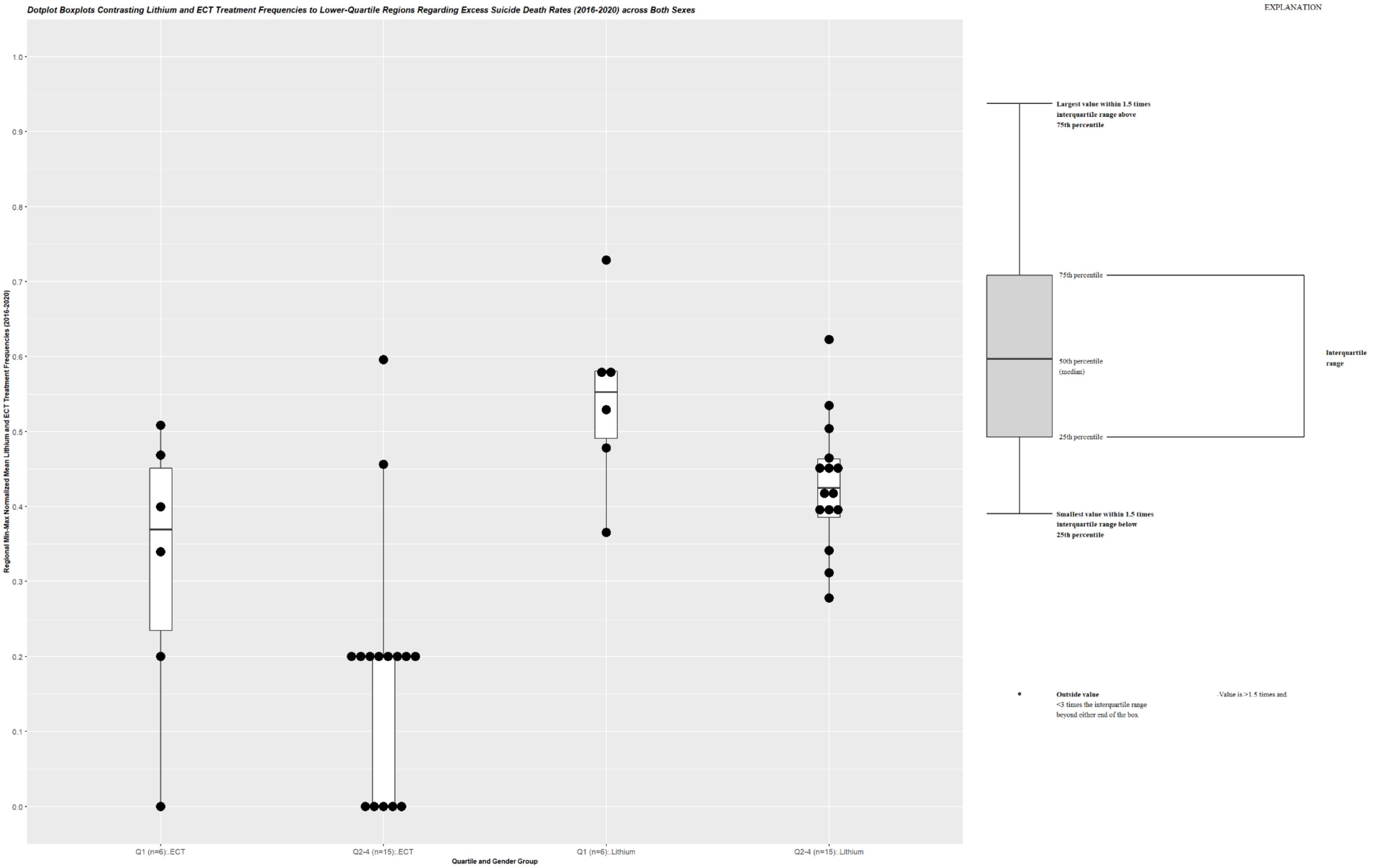

Figure legend: The Y-axis depicts regional values of min-max normalized ECT and lithium treatment usage frequencies averaged across 2016-2020 (derived in Section 2.1 (1)). The X-axis depicts the lower (Q1) and other (Q2-Q4) counties regarding mean of min-max normalized year-wise differences in suicide deaths per 100,000 inhabitants between adolescence and young adulthood (variables derived in Section 2.1 (1) – illustrating only the combined gender group. Thus, per definition, Q1-counties exhibited lower excess adolescent suicide deaths in comparison to Q2-Q4. Treatment usage frequencies were compared between the in-silico generated subgroups in using the one-sided Wilcoxon rank sum exact test. Lower-quartile counties regarding excess adolescent suicide deaths were associated with higher mean treatment usage frequencies of lithium (W=71, p-value=0.022, Bonferroni-adjusted p-value=0.045; Q1 - minima: 0.365, maxima: 0.729, centre [median]: 0.553, bounds of box: 0.491-0.581, lower whisker: 0.494, upper whisker: 0.581; Q2-Q4 - minima: 0.278, maxima: 0.623, centre [median]: 0.425, bounds of box: 0.385-0.464, lower whisker: 0.278, upper whisker: 0.535), whereas ECT exhibited a non-significant trend in the same direction (W=65.5, p-value=0.05, Bonferroni-adjusted p-value=0.101; Q1 - minima: 0, maxima: 0.508, centre [median]: 0.369, bounds of box: 0.235-0.451, lower whisker: 0, upper whisker: 0.508; Q2-Q4 - minima: 0, maxima: 0.596, centre [median]: 0.2, bounds of box: 0-0.2, lower whisker: 0, upper whisker: 0.456) - i.e., indicating that regions with lower excess adolescent suicide death rates exhibited higher lithium and ECT usage frequencies.

Abbreviations: ECT, electroconvulsive therapy; Q1, first quartile; Q2-Q4, second, third and fourth quartile.

Supplementary Figure 3. ECT Usage Frequencies and Regional Excess Suicide Deaths in Females (2016-2020)

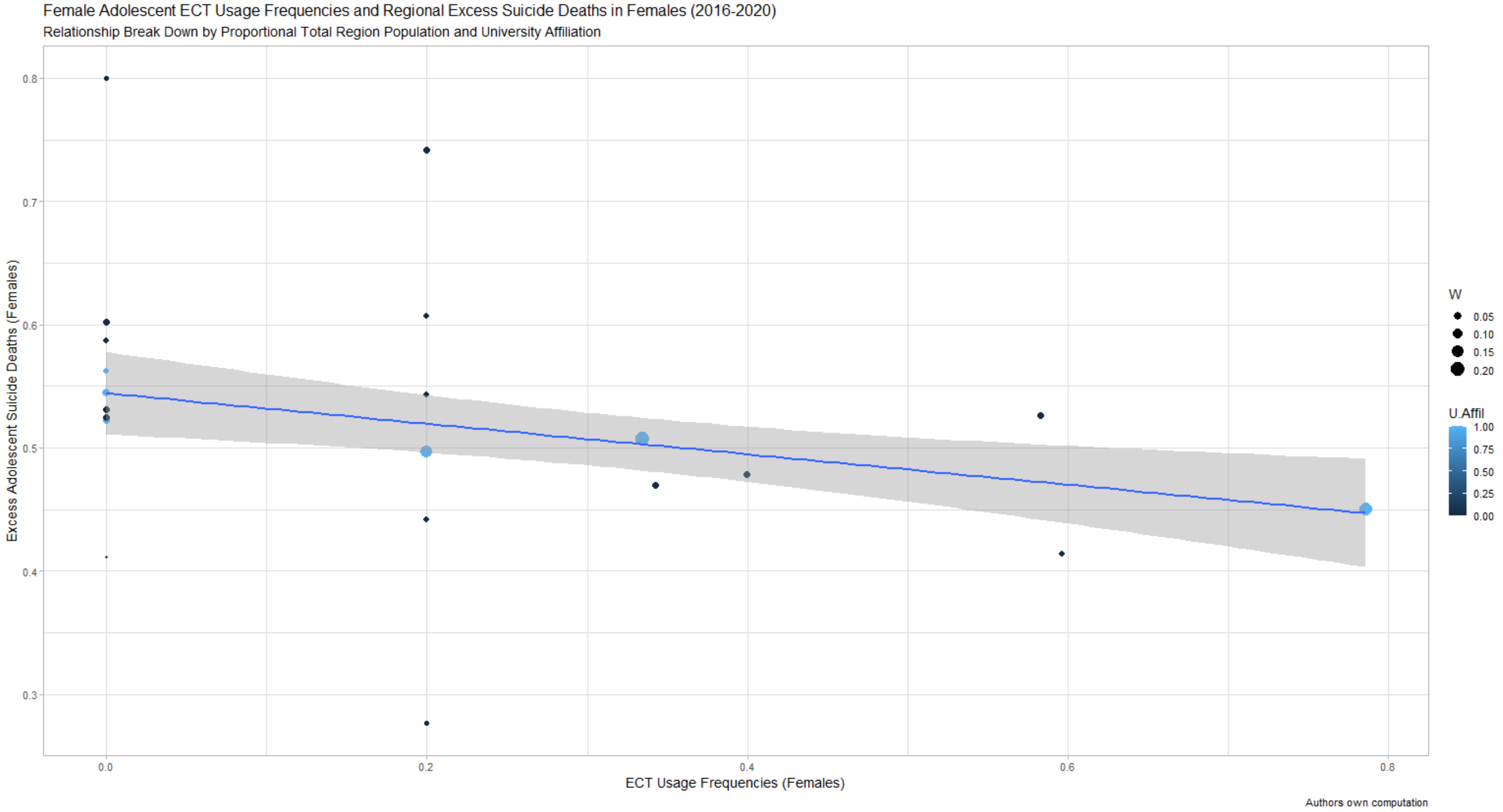

Figure legend: The Y-axis depicts the regional mean of min-max normalized year-wise differences in female suicide deaths per 100,000 inhabitants between adolescence and young adulthood (variables derived in Section 2.1 (1). The X-axis depicts regional mean values of min-max normalized ECT treatment usage frequencies in females across 2016-2020 (derived in Section 2.1 (1)) – subsequently transformed for normalization purposes by Blom's method. The slope and confidence intervals (CI:s) of the robust linear regression model contrasting these two variables are depicted as a blue line (slope) with grey shading (CI:s). Region population in relation to the national population are illustrated by the circle diameter and counties affiliated with medical Universities are highlighted in blue (i.e., Skåne region – Lund University, Stockholm region – Karolinska Institutet, Uppsala region – Uppsala University, Västerbotten region – Umeå University, Västra Götaland region – Gothenburg University/Sahlgrenska Academy). The figures demonstrate that ECT usage frequencies across 2016-2020 are inversely correlated with regional excess adolescent suicide deaths in females (β = -0.613, p-value=0.005, Bonferroni-adjusted p-value=0.016, multiple R-squared: 0.148, adjusted R-squared: 0.104, 95% CI: -0.014, -0.062).

Abbreviations: 95% CI, 95% confidence interval; ECT, electroconvulsive therapy; U.Affil, medical university affiliation (regions affiliated to medical universities are coloured in blue and regions unaffiliated to medical universities are coloured in black); W, weights (regional population size expressed as a percentage of the total national population).

Supplementary Figure 4. Dotplot Boxplots Depicting ECT Usage Frequencies and Regional Excess Suicide Deaths in Females and Males, respectively (2016-2020)

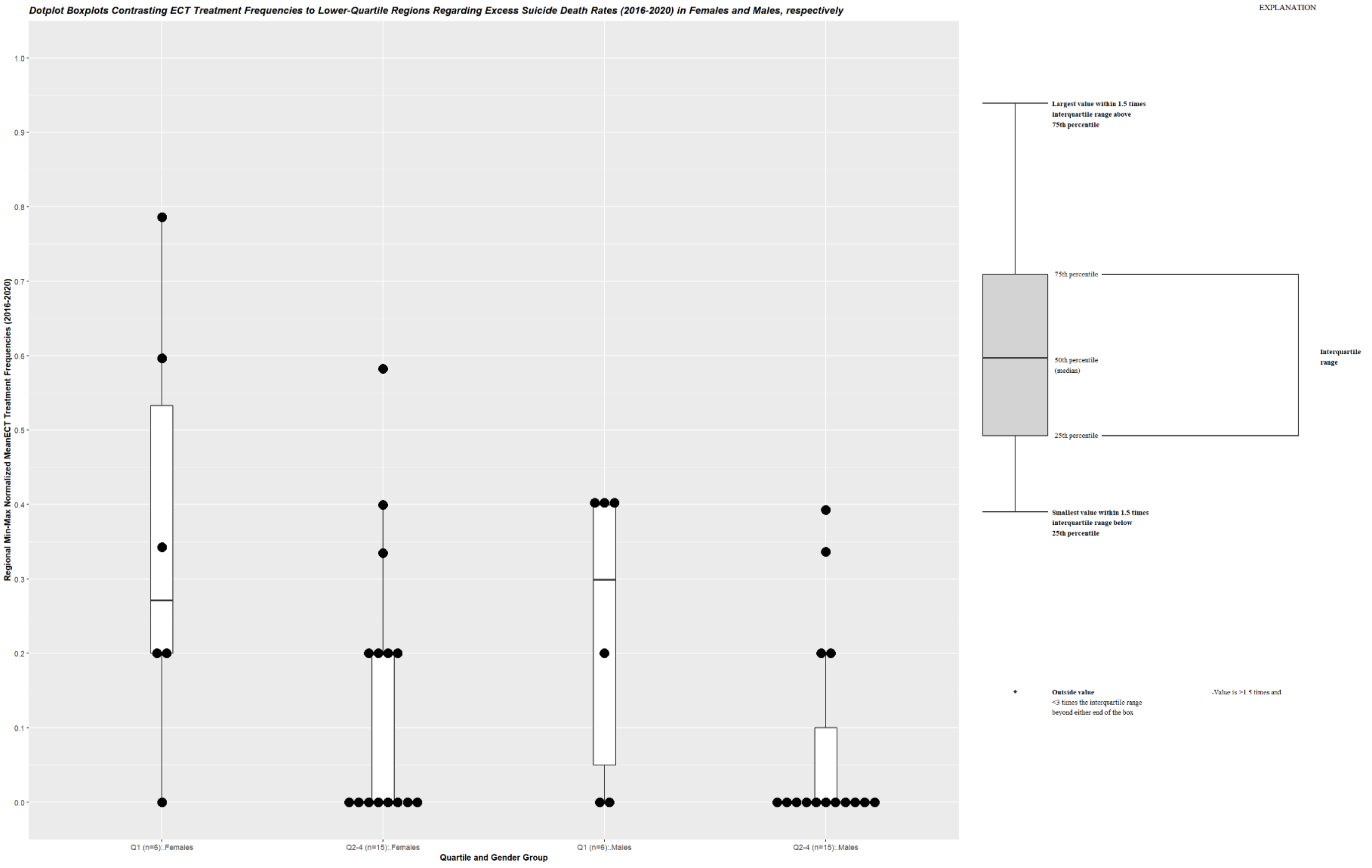

Figure legend: The Y-axis depicts regional values of min-max normalized ECT treatment usage frequencies averaged across 2016-2020 (derived in Section 2.1 (1)) in females and males, respectively. The X-axis depicts the lower (Q1) and other (Q2-Q4) counties regarding mean of min-max normalized year-wise differences in suicide deaths per 100,000 inhabitants between adolescence and young adulthood (variables derived in Section 2.1 (1) – illustrating values for females and males, respectively. Thus, per definition, Q1-counties exhibited lower excess adolescent suicide deaths in comparison to Q2-Q4. Treatment usage frequencies were compared between the in-silico generated subgroups in using the one-sided Wilcoxon rank sum exact test. Lower-quartile counties regarding excess adolescent suicide deaths were associated with higher mean treatment usage frequencies of ECT in females (W=67, p=0.039; Q1 - minima: 0, maxima: 0.786, centre [median]: 0.271, bounds of box: 0.2-0.533, lower whisker: 0, upper whisker: 0.786; Q2-Q4 - minima: 0, maxima: 0.583, centre [median]: 0, bounds of box: 0-0.2, lower whisker: 0, upper whisker: 0.399) and males (W=68, p-value=0.022; Q1 - minima: 0, maxima: 0, centre [median]: 0.299, bounds of box: 0.05-0.399, lower whisker: 0, upper whisker: 0.406; Q2-Q4 - minima: 0, maxima: 0.393, centre [median]: 0, bounds of box: 0-0.1, lower whisker: 0, upper whisker: 0.2), respectively - i.e., indicating that regions with lower excess adolescent suicide death rates exhibited higher ECT usage frequencies.

Abbreviations: ECT, electroconvulsive therapy; Q1, first quartile; Q2-Q4, second, third and fourth quartile.

R output (excluding Figures)

```
[1] "Key-words: (1) B - Both Sexes, (2) F - Females, (3) M - Males, (4) Diff - proxy variable measuring excess adolescent suicide death rates (variables derived in Section 2.1 (1)) (5) LiECTKloz_med - Min-Max Normalized Mean Clozapine, Lithium and ECT Treatment Frequencies across 2016-2020 (derived in Section 2.1 (1))"

[1] "R version\342\200\235)\nprint(\342\200\234Distribution of data - shapiro wilk's tests"


      Shapiro-Wilk normality test


data:  Diff_B

W = 0.9724, p-value = 0.7853




      Shapiro-Wilk normality test


data:  LiECTKloz_med_B

W = 0.96858, p-value = 0.7014




      Shapiro-Wilk normality test


data:  Diff_M

W = 0.97362, p-value = 0.8111
```

```
	Shapiro-Wilk normality test

data:  LiECTKloz_med_M
W = 0.95367, p-value = 0.3987


	Shapiro-Wilk normality test

data:  Diff_F
W = 0.93821, p-value = 0.2008


	Shapiro-Wilk normality test

data:  LiECTKloz_med_F
W = 0.97182, p-value = 0.773


Call:
lm(formula = LiECTKloz_med_B ~ Cloz_B + Lithium_B + ECT_B)
```

```
Residuals:
       Min         1Q     Median         3Q        Max 
-5.054e-16 -2.000e-16 -3.652e-17  2.229e-16  6.770e-16 

Coefficients:
             Estimate Std. Error   t value Pr(>|t|)    
(Intercept) 2.423e-17  3.744e-16 6.500e-02    0.949    
Cloz_B      3.333e-01  5.120e-16 6.510e+14   <2e-16 ***
Lithium_B   3.333e-01  9.375e-16 3.556e+14   <2e-16 ***
ECT_B       3.333e-01  5.584e-16 5.969e+14   <2e-16 ***
---
Signif. codes:  0 '***' 0.001 '**' 0.01 '*' 0.05 '.' 0.1 ' ' 1

Residual standard error: 3.554e-16 on 17 degrees of freedom
Multiple R-squared:      1,	Adjusted R-squared:      1 
F-statistic: 8.446e+29 on 3 and 17 DF,  p-value: < 2.2e-16

[1] 1

Call:
lm(formula = LiECTKloz_med_F ~ Cloz_F + Lithium_F + ECT_F)

Residuals:
```

```
      Min        1Q    Median        3Q       Max

-0.082648 -0.023294 -0.003746  0.022620  0.072557


Coefficients:

            Estimate Std. Error t value Pr(>|t|)

(Intercept)  0.02511    0.03775   0.665  0.51493

Cloz_F       0.40879    0.05298   7.715 5.96e-07 ***

Lithium_F    0.31243    0.08605   3.631  0.00207 **

ECT_F        0.23726    0.04462   5.318 5.67e-05 ***

---

Signif. codes:  0 '***' 0.001 '**' 0.01 '*' 0.05 '.' 0.1 ' ' 1


Residual standard error: 0.04087 on 17 degrees of freedom

Multiple R-squared:  0.9164,     Adjusted R-squared:  0.9016

F-statistic: 62.09 on 3 and 17 DF,  p-value: 2.284e-09


[1] 1.308421


Call:

lm(formula = LiECTKloz_med_M ~ Cloz_M + Lithium_M + ECT_M)


Residuals:

      Min        1Q    Median        3Q       Max
```

```
-0.145097 -0.046935  0.007113  0.039283  0.166199


Coefficients:

            Estimate Std. Error t value Pr(>|t|)

(Intercept)  0.13945    0.04336   3.216  0.00507 **

Cloz_M       0.19119    0.12081   1.583  0.13194

Lithium_M    0.17767    0.13743   1.293  0.21335

ECT_M        0.35639    0.12010   2.967  0.00863 **

---

Signif. codes:  0 '***' 0.001 '**' 0.01 '*' 0.05 '.' 0.1 ' ' 1


Residual standard error: 0.08011 on 17 degrees of freedom

Multiple R-squared:  0.6544,     Adjusted R-squared:  0.5934

F-statistic: 10.73 on 3 and 17 DF,  p-value: 0.0003413


[1] 1.864062

[1] "###1.2.1 Main robust linear regression model - Both Sexes"


Call:

lmrob(formula = Diff_B ~ LiECTKloz_med_B, weights = W, method = "MM", setting = "KS2014")

 \--> method = "MM"

Residuals:

     Min       1Q   Median       3Q      Max
```

```
-0.19050 -0.04823 -0.01021  0.03805  0.16011


Coefficients:
                Estimate Std. Error t value Pr(>|t|)
(Intercept)      0.71514    0.05775  12.383 1.52e-10 ***
LiECTKloz_med_B -0.61324    0.14050  -4.365 0.000333 ***
---
Signif. codes:  0 '***' 0.001 '**' 0.01 '*' 0.05 '.' 0.1 ' ' 1


Robust residual standard error: 0.01459
Multiple R-squared:  0.1229,     Adjusted R-squared:  0.07669
Convergence in 14 IRWLS iterations


Robustness weights:
 14 weights are ~= 1. The remaining 7 ones are
     7      8     10     16     19     20     21
0.9154 0.8151 0.9974 0.9919 0.8455 0.9481 0.4382
Algorithmic parameters:
      tuning.chi1       tuning.chi2       tuning.chi3       tuning.chi4
       -5.000e-01         1.500e+00                NA         5.000e-01
               bb       tuning.psi1       tuning.psi2       tuning.psi3
        5.000e-01        -5.000e-01         1.500e+00         9.500e-01
      tuning.psi4        refine.tol           rel.tol         scale.tol
```

```
               NA         1.000e-07         1.000e-07         1.000e-10
        solve.tol       eps.outlier             eps.x warn.limit.reject
        1.000e-07         4.762e-03         8.705e-13         5.000e-01
warn.limit.meanrw
        5.000e-01
     nResample         max.it       best.r.s       k.fast.s          k.max
          1000            500             20              2           2000
   maxit.scale      trace.lev            mts     compute.rd fast.s.large.n
           200              0           1000              0           2000
              setting                    psi            subsampling
             "KS2014"                  "lqq"          "nonsingular"
                  cov compute.outlier.stats
            ".vcov.w"                  "SM"
seed : int(0)
                     2.5 %     97.5 %
(Intercept)      0.6019479  0.8283397
LiECTKloz_med_B -0.8886105 -0.3378689
[1] "###1.3.1. Main robust linear regression model - Females"


Call:
lmrob(formula = Diff_F ~ LiECTKloz_med_F, weights = W, method = "MM", setting = "KS2014")
 \--> method = "MM"
Residuals:
```

```
      Min        1Q    Median        3Q       Max
-0.255713 -0.033873  0.007458  0.044114  0.250853

Coefficients:
                Estimate Std. Error t value Pr(>|t|)
(Intercept)      0.56289    0.04463  12.613 1.11e-10 ***
LiECTKloz_med_F -0.14855    0.11108  -1.337    0.197
---
Signif. codes:  0 '***' 0.001 '**' 0.01 '*' 0.05 '.' 0.1 ' ' 1

Robust residual standard error: 0.01435
Multiple R-squared:  0.05891,    Adjusted R-squared:  0.009381
Convergence in 7 IRWLS iterations

Robustness weights:
 16 weights are ~= 1. The remaining 5 ones are
     1      6      8     17     21
0.6463 0.7469 0.9936 0.5362 0.9692
Algorithmic parameters:
      tuning.chi1       tuning.chi2       tuning.chi3       tuning.chi4
       -5.000e-01         1.500e+00                NA         5.000e-01
               bb       tuning.psi1       tuning.psi2       tuning.psi3
        5.000e-01        -5.000e-01         1.500e+00         9.500e-01
```

```
      tuning.psi4        refine.tol           rel.tol         scale.tol
               NA         1.000e-07         1.000e-07         1.000e-10
        solve.tol       eps.outlier             eps.x warn.limit.reject
        1.000e-07         4.762e-03         8.705e-13         5.000e-01
warn.limit.meanrw
        5.000e-01
     nResample         max.it       best.r.s       k.fast.s          k.max
          1000            500             20              2           2000
   maxit.scale      trace.lev            mts     compute.rd fast.s.large.n
           200              0           1000              0           2000
              setting                   psi            subsampling
             "KS2014"                 "lqq"          "nonsingular"
                  cov compute.outlier.stats
            ".vcov.w"                  "SM"
seed : int(0)
                     2.5 %     97.5 %
(Intercept)      0.5133803  0.7245721
LiECTKloz_med_M -0.6784449 -0.1296751
[1] "1.4.1. Main robust linear regression model - Males"


Call:
lmrob(formula = Diff_M ~ LiECTKloz_med_M, weights = W, method = "MM", setting = "KS2014")
 \--> method = "MM"
```

```
Residuals:
       Min         1Q     Median         3Q        Max 
-0.1921975 -0.0425645 -0.0009729  0.0734401  0.1772256 


Coefficients:
                Estimate Std. Error t value Pr(>|t|)    
(Intercept)      0.61898    0.05388  11.489 5.39e-10 ***
LiECTKloz_med_M -0.40406    0.13999  -2.886  0.00946 ** 
---
Signif. codes:  0 '***' 0.001 '**' 0.01 '*' 0.05 '.' 0.1 ' ' 1


Robust residual standard error: 0.01543 
Multiple R-squared:  0.01782,	Adjusted R-squared:  -0.03388 
Convergence in 11 IRWLS iterations


Robustness weights: 
 13 weights are ~= 1. The remaining 8 ones are
     7     11     12     15     16     17     18     20 
0.9793 0.9637 0.9898 0.9565 0.6561 0.8441 0.7265 0.8453 
Algorithmic parameters: 
      tuning.chi1       tuning.chi2       tuning.chi3       tuning.chi4 
       -5.000e-01         1.500e+00                NA         5.000e-01 
               bb       tuning.psi1       tuning.psi2       tuning.psi3 
```

```
        5.000e-01        -5.000e-01         1.500e+00         9.500e-01
      tuning.psi4        refine.tol           rel.tol         scale.tol
               NA         1.000e-07         1.000e-07         1.000e-10
        solve.tol       eps.outlier             eps.x warn.limit.reject
        1.000e-07         4.762e-03         8.705e-13         5.000e-01
warn.limit.meanrw
        5.000e-01
     nResample         max.it       best.r.s       k.fast.s          k.max
          1000            500             20              2           2000
   maxit.scale      trace.lev            mts     compute.rd fast.s.large.n
           200              0           1000              0           2000
              setting                   psi           subsampling
             "KS2014"                 "lqq"         "nonsingular"
                  cov compute.outlier.stats
            ".vcov.w"                  "SM"
seed : int(0)
                     2.5 %     97.5 %
(Intercept)      0.5133803  0.7245721
LiECTKloz_med_M -0.6784449 -0.1296751


      Wilcoxon rank sum exact test


data:  LiECTKloz_med_B by Diff_B_d
```

```
W = 67, p-value = 0.04742
alternative hypothesis: true location shift is greater than 0



	Wilcoxon rank sum exact test

data:  LiECTKloz_med_M by Diff_M_d
W = 74, p-value = 0.01167
alternative hypothesis: true location shift is greater than 0

[1] 0.0163659
[1] 0.8673317
[1] 0.01135405
[1] 0.001888864
[1] 0.2269925
[1] 0.001417475
[1] 0.008757057
[1] 0.1266691
[1] 2.169314e-05
      cor
0.3426656
      cor
0.2908462
```

```
      cor
0.6155726
      cor
0.3282221
        cor
-0.01619236
      cor
0.3032756
      cor
0.2003222
      cor
0.6489056
      cor
0.4223985

	Shapiro-Wilk normality test

data:  Avg_ECT_B_Lithium_B
W = 0.91257, p-value = 0.06173

[1] 0.5048153
                        Estimate Std. Error   t value     Pr(>|t|)
(Intercept)          0.54114558 0.02546711 21.248800 3.377056e-14
```

```
blom(Cloz_B)        -0.03549536 0.02564899 -1.383889 1.833138e-01

Avg_ECT_B_Lithium_B -0.13601651 0.04188744 -3.247191 4.473002e-03

[1] 0.1006289

Warning message:

In wilcox.test.default(x = c(0.399246615047065, 0, 0.339596480444415,  :

  cannot compute exact p-value with ties

[1] 0.04485478

[1] 2.195206

[1] 0.9036281

[1] 0.0159148

                  2.5 %      97.5 %

(Intercept)  0.49854592  0.54640938

blom(ECT_F) -0.06150621 -0.01429858


      Wilcoxon rank sum test with continuity correction


data:  ECT_F by Diff_F_d

W = 67, p-value = 0.03879

alternative hypothesis: true location shift is greater than 0


Warning message:

In wilcox.test.default(x = c(0.2, 0, 0.342578437688951, 0.2, 0.596322053065887,  :

  cannot compute exact p-value with ties
```

```
Call:
lmrob(formula = Diff_M ~ blom(ECT_M) + Avg_Cloz_M_Lithium_M, weights = W,
    method = "MM", setting = "KS2014")
 \--> method = "MM"
Residuals:
     Min       1Q   Median       3Q      Max
-0.23103 -0.04738  0.00469  0.04014  0.18848

Coefficients:
                     Estimate Std. Error t value Pr(>|t|)
(Intercept)           0.51388    0.05426   9.471 2.05e-08 ***
blom(ECT_M)          -0.05763    0.02171  -2.654   0.0161 *
Avg_Cloz_M_Lithium_M -0.01780    0.13207  -0.135   0.8943
---
Signif. codes:  0 '***' 0.001 '**' 0.01 '*' 0.05 '.' 0.1 ' ' 1

Robust residual standard error: 0.01513
Multiple R-squared:  0.06652,    Adjusted R-squared:  -0.0372
Convergence in 11 IRWLS iterations

Robustness weights:
 16 weights are ~= 1. The remaining 5 ones are
```

```
     7     12     16     17     18
0.9159 0.8782 0.5786 0.8979 0.5384
Algorithmic parameters:
      tuning.chi1       tuning.chi2       tuning.chi3       tuning.chi4
       -5.000e-01         1.500e+00                NA         5.000e-01
               bb       tuning.psi1       tuning.psi2       tuning.psi3
        5.000e-01        -5.000e-01         1.500e+00         9.500e-01
      tuning.psi4        refine.tol           rel.tol         scale.tol
               NA         1.000e-07         1.000e-07         1.000e-10
        solve.tol       eps.outlier             eps.x warn.limit.reject
        1.000e-07         4.762e-03         1.655e-12         5.000e-01
warn.limit.meanrw
        5.000e-01
     nResample         max.it       best.r.s       k.fast.s          k.max
          1000            500             20              2           2000
   maxit.scale      trace.lev            mts     compute.rd fast.s.large.n
           200              0           1000              0           2000
              setting                     psi            subsampling
             "KS2014"                   "lqq"          "nonsingular"
                  cov compute.outlier.stats
           ".vcov.w"                   "SM"
seed : int(0)
```

```
	Wilcoxon rank sum test with continuity correction

data:  ECT_M by Diff_M_d
W = 68, p-value = 0.02233
alternative hypothesis: true location shift is greater than 0


Warning message:
In wilcox.test.default(x = c(0.399319704375601, 0, 0.2, 0.406397458751859,  :
  cannot compute exact p-value with ties
`geom_smooth()` using formula 'y ~ x'
`geom_smooth()` using formula 'y ~ x'
Warning message:
Ignoring unknown parameters: binwith
`stat_bindot()` using `bins = 30`. Pick better value with `binwidth`.
`stat_bindot()` using `bins = 30`. Pick better value with `binwidth`.
        ymin     lower    middle     upper      ymax               outliers
1 0.35594542 0.3754555 0.4461262 0.4773353 0.5323499              0.1216931
2 0.12816537 0.2642307 0.2880741 0.3593158 0.3756139 0.09252874, 0.55791571
3 0.12169312 0.2389182 0.3938706 0.4572394 0.5145373
4 0.09252874 0.2319661 0.2800000 0.3567553 0.4343493              0.5486764
5 0.29485747 0.3308043 0.3718247 0.4557594 0.5064423
6 0.09251701 0.1674285 0.2545156 0.3316700 0.5122629
  notchupper notchlower x flipped_aes PANEL group ymin_final ymax_final xmin
```

```
1  0.5118419  0.3804104 1       FALSE     1     1 0.12169312  0.5323499 0.95
2  0.3268644  0.2492837 2       FALSE     1     2 0.09252874  0.5579157 1.95
3  0.5346948  0.2530464 3       FALSE     1     3 0.12169312  0.5145373 2.95
4  0.3309083  0.2290917 4       FALSE     1     4 0.09252874  0.5486764 3.95
5  0.4524248  0.2912247 5       FALSE     1     5 0.29485747  0.5064423 4.95
6  0.3215186  0.1875126 6       FALSE     1     6 0.09251701  0.5122629 5.95
  xmax xid newx new_width weight colour  fill size alpha shape linetype
1 1.05   1    1       0.1      1 grey20 white  0.5    NA    19    solid
2 2.05   2    2       0.1      1 grey20 white  0.5    NA    19    solid
3 3.05   3    3       0.1      1 grey20 white  0.5    NA    19    solid
4 4.05   4    4       0.1      1 grey20 white  0.5    NA    19    solid
5 5.05   5    5       0.1      1 grey20 white  0.5    NA    19    solid
6 6.05   6    6       0.1      1 grey20 white  0.5    NA    19    solid
Warning message:
Ignoring unknown parameters: binwith
`stat_bindot()` using `bins = 30`. Pick better value with `binwidth`.
`stat_bindot()` using `bins = 30`. Pick better value with `binwidth`.
       ymin     lower    middle     upper      ymax  outliers notchupper
1 0.0000000 0.2348991 0.3694215 0.4511703 0.5084926             0.5089235
2 0.0000000 0.0000000 0.2000000 0.2000000 0.4558783 0.5957549  0.2815908
3 0.3650794 0.4906439 0.5525098 0.5806102 0.5821229 0.7285714  0.6105410
4 0.2775862 0.3854540 0.4247788 0.4636033 0.5346535 0.6228571  0.4566601
  notchlower x flipped_aes PANEL group ymin_final ymax_final xmin xmax xid newx
```

```
1  0.2299196 1       FALSE     1     1  0.0000000  0.5084926 0.95 1.05   1    1
2  0.1184092 2       FALSE     1     2  0.0000000  0.5957549 1.95 2.05   2    2
3  0.4944786 3       FALSE     1     3  0.3650794  0.7285714 2.95 3.05   3    3
4  0.3928974 4       FALSE     1     4  0.2775862  0.6228571 3.95 4.05   4    4
  new_width weight colour  fill size alpha shape linetype
1       0.1      1 grey20 white  0.5    NA    19    solid
2       0.1      1 grey20 white  0.5    NA    19    solid
3       0.1      1 grey20 white  0.5    NA    19    solid
4       0.1      1 grey20 white  0.5    NA    19    solid
`geom_smooth()` using formula 'y ~ x'
Warning message:
Ignoring unknown parameters: binwith
`stat_bindot()` using `bins = 30`. Pick better value with `binwidth`.
`stat_bindot()` using `bins = 30`. Pick better value with `binwidth`.
  ymin lower    middle     upper      ymax             outliers notchupper
1    0  0.20 0.2712892 0.5328861 0.7857249                      0.48601154
2    0  0.00 0.0000000 0.2000000 0.3992715            0.5825385 0.08159085
3    0  0.05 0.2988517 0.3989156 0.4063975                      0.52391358
4    0  0.00 0.0000000 0.1000000 0.2000000 0.3363625, 0.3927088 0.04079542
   notchlower x flipped_aes PANEL group ymin_final ymax_final xmin xmax xid
1  0.05656690 1       FALSE     1     1          0  0.7857249 0.95 1.05   1
2 -0.08159085 2       FALSE     1     2          0  0.5825385 1.95 2.05   2
3  0.07378986 3       FALSE     1     3          0  0.4063975 2.95 3.05   3
```

```
4 -0.04079542 4       FALSE     1     4          0  0.3927088 3.95 4.05   4
  newx new_width weight colour  fill size alpha shape linetype
1    1       0.1      1 grey20 white  0.5    NA    19    solid
2    2       0.1      1 grey20 white  0.5    NA    19    solid
3    3       0.1      1 grey20 white  0.5    NA    19    solid
4    4       0.1      1 grey20 white  0.5    NA    19    solid
```

References (Supplementary Methods)

1. Nordenskjöld A. *Årsrapport 2020.*; 2020. https://registercentrum.blob.core.windows.net/ect/r/Kvalitetsregister-ECT-A-rsrapport-2020-utan-PAR-Hkgpr3QIj_.pdf
2. Todorov V, Filzmoser P. An Object-Oriented Framework for Robust Multivariate Analysis. *J Stat Softw*. 2010;32(3):1-47. doi:10.18637/JSS.V032.I03
3. Mangiafico S. rcompanion: Functions to Support Extension Education Program Evaluation. R package version 2.4.15, <https://CRAN.R-project.org/package=rcompanion>. Published online 2022.
4. Maechler M, Rousseeuw P, Croux C, et al. robustbase: Basic Robust Statistics R Package version 0.95-0. Published 2022. http://cran.r-project.org/package=robustbase
5. Yohai VJ. High Breakdown-Point and High Efficiency Robust Estimates for Regression. *https://doi.org/101214/aos/1176350366*. 1987;15(2):642-656. doi:10.1214/AOS/1176350366
6. Wickham H. ggplot2: Elegant Graphics for Data Analysis. Published online 2016.
7. DeCicco L. Exploring ggplot2 boxplots - Defining limits and adjusting style | Water Data For The Nation Blog. Accessed December 6, 2022. https://waterdata.usgs.gov/blog/boxplots/
8. Soloman SR, Sawilowsky SS. Impact of Rank-Based Normalizing Transformations on the Accuracy of Test Scores. *J Mod Appl Stat Methods*. 2009;8(2):448-462. doi:10.22237/jmasm/1257034080
9. Armstrong RA. When to use the Bonferroni correction. *Ophthalmic Physiol Opt*. 2014;34(5):502-508. doi:10.1111/OPO.12131
