# Supplementary material for: Regional clozapine, ECT and lithium usage inversely associated with excess suicide rates in male adolescents
Source: Nat Commun. 2023 Mar 14;14:1281. doi: 10.1038/s41467-023-36973-4 (PMC10015020; doi:10.1038/s41467-023-36973-4)
Supplement: Supplementary file 3 — Reporting Summary [file 41467_2023_36973_MOESM3_ESM.pdf]

nature portfolio

Corresponding author(s): Dr. Adrian Emmanuel Desai Boström

Last updated by author(s): 07/12/2022

Reporting Summary

Nature Portfolio wishes to improve the reproducibility of the work that we publish. This form provides structure for consistency and transparency in reporting. For further information on Nature Portfolio policies, see our Editorial Policies and the Editorial Policy Checklist.

Statistics

For all statistical analyses, confirm that the following items are present in the figure legend, table legend, main text, or Methods section.

n/a	Confirmed	
☐	☒	The exact sample size (n) for each experimental group/condition, given as a discrete number and unit of measurement
☐	☒	A statement on whether measurements were taken from distinct samples or whether the same sample was measured repeatedly
☐	☒	The statistical test(s) used AND whether they are one- or two-sided<br>*Only common tests should be described solely by name; describe more complex techniques in the Methods section.*
☐	☒	A description of all covariates tested
☐	☒	A description of any assumptions or corrections, such as tests of normality and adjustment for multiple comparisons
☐	☒	A full description of the statistical parameters including central tendency (e.g. means) or other basic estimates (e.g. regression coefficient) AND variation (e.g. standard deviation) or associated estimates of uncertainty (e.g. confidence intervals)
☐	☒	For null hypothesis testing, the test statistic (e.g. F, t, r) with confidence intervals, effect sizes, degrees of freedom and P value noted<br>*Give P values as exact values whenever suitable.*
☒	☐	For Bayesian analysis, information on the choice of priors and Markov chain Monte Carlo settings
☐	☒	For hierarchical and complex designs, identification of the appropriate level for tests and full reporting of outcomes
☐	☒	Estimates of effect sizes (e.g. Cohen's d, Pearson's r), indicating how they were calculated

Our web collection on statistics for biologists contains articles on many of the points above.

Software and code

Policy information about availability of computer code

Data collection	All data was openly available and collected from the Swedish National Board of Health and Welfare and the Swedish ECT registry. This data was extracted from the websites into Microsoft Excel365 MSO [Version 2210 Build 16.0.15726.20188, 64-bit]. Links from which these data can be collected for replication purposes are provided in the Methods section 'Data Sources and Initial Processing.
Data analysis	Raw and processed data generated in this study have been deposited in its entirety in the Open Science Framework (OSF) repository, openly accessible through the following web-links: https://osf.io/ktn5a (raw and processed data, written for Microsoft Excel365 MSO [Version 2210 Build 16.0.15726.20188] 64-bit) and https://osf.io/qtvey (R-code, written for R version 4.0.3).

For manuscripts utilizing custom algorithms or software that are central to the research but not yet described in published literature, software must be made available to editors and reviewers. We strongly encourage code deposition in a community repository (e.g. GitHub). See the Nature Portfolio guidelines for submitting code & software for further information.

Data

Policy information about availability of data

All manuscripts must include a data availability statement. This statement should provide the following information, where applicable:

- Accession codes, unique identifiers, or web links for publicly available datasets
- A description of any restrictions on data availability
- For clinical datasets or third party data, please ensure that the statement adheres to our policy

Raw and processed data generated in this study have been deposited in its entirety in the Open Science Framework (OSF) repository, openly accessible through this web-link: https://osf.io/ktn5a (raw and processed data, written for Microsoft Excel365 MSO [Version 2210 Build 16.0.15726.20188] 64-bit). This data was retrieved from openly accessible data repositories provided by the Swedish National Board of Health and Welfare and the Swedish ECT registry. Web links from which the data can be collected from its original sources for replication or other research purposes are provided in Methods section - 'Data Sources and Initial Processing'. The analysis code has been deposited to the Open Science Framework (OSF) repository, openly accessible through this web-link: https://osf.io/j5zd6 (R-code, written for R version 4.2.0).

Human research participants

Policy information about studies involving human research participants and Sex and Gender in Research.

Reporting on sex and gender	This manuscript uses the term sex (biological attribute) to refer to assigned sex (female or male). Analyses and findings are investigated across both sexes and in males and females, separately. Baseline data for each gender (and combined across all sexes) is openly available, readily accessible and detailed in the Data Availability Statement. No individual-level data is reported. Sex-based post-hoc analyses were performed and are extensively discussed throughout the manuscript.
Population characteristics	Available population characteristics are extensively reported throughout the manuscript (age groups and sexes studied).
Recruitment	The study did not involve recruitment of any patients.
Ethics oversight	The study pertained to openly-accessible data reported by Swedish National Registers. Thus, formal ethical approval from relevant authorities was not considered applicable.

Note that full information on the approval of the study protocol must also be provided in the manuscript.

Field-specific reporting

Please select the one below that is the best fit for your research. If you are not sure, read the appropriate sections before making your selection.

☐ Life sciences ☐ Behavioural & social sciences ☒ Ecological, evolutionary & environmental sciences

For a reference copy of the document with all sections, see nature.com/documents/nr-reporting-summary-flat.pdf

Ecological, evolutionary & environmental sciences study design

All studies must disclose on these points even when the disclosure is negative.

Study description	Using real-world Swedish registry data across the 21 Swedish regions (2016-2020) and implementing rigorous procedures to reduce putative confounding, the present study investigated associations between regional excess adolescent suicide death rates and treatment usage frequencies of clozapine, electroconvulsive therapy (ECT) and lithium.
Research sample	The sample consisted of data encompassing all the 21 Swedish regions and included (1) year-wise confirmed suicide death rates and lithium and clozapine treatment usage frequencies (number of recorded dispensations) - as reported to the Swedish National Board of Health and Welfare – and the yearly number of patients receiving ECT treatment - as reported to the National ECT registry (coverage rate ~90%). The full data was retrieved across the age-ranges 15-19 and 20-24, for both sexes and males and females separately. Thus, the sample represents all registered Swedish citizens that in the years 2016-2020 were in adolescence (15-19 years old) or young adulthood (20-24 years old).
Sampling strategy	The sample size consisted of the 21 Swedish regions, that were compared. Sample size was selected to comprehensively include all the regions. For linear regressions, i.e. multivariable analyses for which the outcome variable is numerical, it is necessary to have at least 10 observations per co-variate. The main results of this study included only 1 co-variate, relying on variabel normalization to reduce putative confounding. Individual regressions were modeled on two co-variates, which would also satisfy this criteria (i.e., n=21 > 10 x2). The sample size was thus considered sufficient for the intended analyses.
Data collection	All data was openly available and collected from the Swedish National Board of Health and Welfare and the Swedish ECT registry. Data from the Swedish ECT registry [regional year-wise ECT utilization frequencies in < 18-year-olds] was manually recorded into Microsoft Excel365 MSO [Version 2210 Build 16.0.15726.20188, 64-bit] by A.E.D.B.. P.A. performed the same extraction for validation purposes. All other data was downloaded from the provided Swedish National Board of Health and Welfare weblinks and were

	imported into Microsoft Excel365 MSO [Version 2210 Build 16.0.15726.20188, 64-bit] by A.E.D.B.. P.A., again, performed the same extraction for validation purposes.
Timing and spatial scale	The time-period was chosen to match the major course direction in national treatment guidelines regarding ECT – since 2016 and with highest priority recommending its use in the care of post-pubertal adolescents with severe MDD with mood-congruent psychotic symptoms, catatonia, or treatment resistance. All data was retrieved 2021 Nov 15. There were no gaps between collection periods, which was collected during the same day. At the time of data-extraction, information on regional suicide death rates was available up to 2020, hence, 2021–2022 treatment frequencies were not included in the analysis. This is further described in Online Methods section - 'Statistical Considerations'.
Data exclusions	No data points were excluded from the analysis.
Reproducibility	To enable the expedient replication of our results, sourced data, analysis code and other important information have been made deposited to readily accessible data depositories. The original analysis was performed by A.E.D.B., and results were fully replicated by M-R.A.. All attempts to repeat the experiment were successful.
Randomization	Data on suicide death rates and CEL-treatment utilization rates were reported for the 21 Swedish regions separately and for the years 2016, 2017, 2018, 2019 and 2020. The allocation of values into groups were performed based on region - i.e., values were investigated separately for each region. Values for regional suicide death rates were subtracted between 15-19-olds and 20-24-year-olds for the years 2016-2020, separately. At this stage, each region presented 4 variables (differential suicide death rates, clozapine, ECT and lithium utilization rates), each with 5 values for the years 2016-2020. These variables were subjected to min-max normalization over the years 2016-2020, separately for each region and variable. The regional mean for min-max normalized clozapine, ECT and lithium utilization rates constituted the variable 'mean clozapine, ECT and lithium treatment usage frequencies', i.e., with one value for each of the 21 Swedish regions (n=21). Similarly, each region was represented by one value for excess adolescent suicide death rates, which constituted the min-max normalized suicide death rates (subtracted between age-groups) over the years 2016-2021, i.e., with one value for each of the 21 Swedish regions (n=21).<br><br>Data availability issues precluded the possibility to explore the potential moderating role on the observed associations of clinical characteristics of individual adolescent recipients of the studied treatment modalities, dosages of lithium or clozapine and the number of ECT treatment courses administered to individual patients. Therefore, randomized allocation of samples into experimental groups was not possible.
Blinding	Data availability issues precluded the possibility to explore the potential moderating role on the observed associations of clinical characteristics of individual adolescent recipients of the studied treatment modalities, dosages of lithium or clozapine and the number of ECT treatment courses administered to individual patients. Therefore, blinding of samples was not possible.

Did the study involve field work? ☐ Yes ☒ No

Reporting for specific materials, systems and methods

We require information from authors about some types of materials, experimental systems and methods used in many studies. Here, indicate whether each material, system or method listed is relevant to your study. If you are not sure if a list item applies to your research, read the appropriate section before selecting a response.

Materials & experimental systems

n/a	Involved in the study
☒	☐ Antibodies
☒	☐ Eukaryotic cell lines
☒	☐ Palaeontology and archaeology
☒	☐ Animals and other organisms
☒	☐ Clinical data
☒	☐ Dual use research of concern

Methods

n/a	Involved in the study
☒	☐ ChIP-seq
☒	☐ Flow cytometry
☒	☐ MRI-based neuroimaging
